# *Vibrio vulnificus* Hemolysin: Biological Activity, Regulation of *vvhA* Expression, and Role in Pathogenesis

**DOI:** 10.3389/fimmu.2020.599439

**Published:** 2020-10-23

**Authors:** Yuan Yuan, Zihan Feng, Jinglin Wang

**Affiliations:** State Key Laboratory of Pathogen and Biosecurity, Beijing Institute of Microbiology and Epidemiology, Academy of Military Medical Sciences (AMMS), Beijing, China

**Keywords:** *Vibrio vulnificus* hemolysin (VVH), cholesterol-dependent cytolysin (CDC), biological activity, gene regulation, sepsis, pathogenesis

## Abstract

The *Vibrio vulnificus* (*V. vulnificus*) hemolysin (VVH) is a pore-forming cholesterol-dependent cytolysin (CDC). Although there has been some debate surrounding the *in vivo* virulence effects of the VVH, it is becoming increasingly clear that it drives different cellular outcomes and is involved in the pathogenesis of *V. vulnificus*. This minireview outlines recent advances in our understanding of the regulation of *vvhA* gene expression, the biological activity of the VVH and its role in pathogenesis. An in-depth examination of the role of the VVH in *V. vulnificus* pathogenesis will help reveal the potential targets for therapeutic and preventive interventions to treat fatal *V. vulnificus* septicemia in humans. Future directions in VVH research will also be discussed.

## Introduction

*V. vulnificus* is an opportunistic human pathogen commonly found in estuarine environments. Human infections usually occur following the consumption of contaminated seafood or *via* an open wound exposed to a contaminated water source (1). Consumption of contaminated raw oysters can result in rapidly fatal septicemia in susceptible individuals, with *V. vulnificus* having the highest fatality rate among all food-borne pathogens (2). However, many aspects related to the biology, genomics, and virulence capabilities of *V. vulnificus* remain elusive or poorly understood (1, 3). During the last decade, research has mainly been focused on the pathogenic mechanisms and virulence factors adopted by *V. vulnificus* (2, 4). The capsule has proven to be a critical virulence factor, with non-encapsulated *V. vulnificus* isogenic mutants readily phagocytosed by host immune cells (5). The *V. vulnificus* multifunctional-auto processing repeats-in-toxin (MARTX) toxin is also likely to be critical to the success of infection. Supporting this, Gavin et al. showed that the MARTX toxin is essential for bacterial dissemination from the intestine (6), while Jones and Oliver demonstrated that the overwhelming tissue destruction that characterizes *V. vulnificus* infections contracted either *via* ingestion or wound infection likely results from the powerful collagenase, metalloproteases, and lipases/phospholipases produced by the bacterium (4). Moreover, MARTX is also known to take part in resistance to phagocytosis, cell destruction, and sepsis (7, 8).

Although the VVH belongs to the cytolytic pore-forming family of toxins (PFTs), all of which cause cytolysis in a variety of mammalian cells, VVH as a virulence factor is under debate. An earlier study has shown that disruption of hemolysin gene *vvhA* had no effect on the virulence of *V. vulnificus* in a mouse lethality model (9). However, other studies have confirmed that *vvhA* gene is substantially regulated and expressed *in vivo* and is likely to play important roles in the pathogenesis of *V. vulnificus* (10, 11). For example, V*. vulnificus* is known for a siderophilic bacterium and iron as one of the factors that regulate *vvhA* expression (12–14). Fan JJ et al. indicate that there was a small difference in mortality when wild type and *vvhA*-deficient mutant strains were force-fed to mice, but VVH seemed to be important for causing damage in the alimentary tract of the mice (15). Moreover, another study suggested that in addition to the MARTX toxin, the VVH may contribute to bacterial invasion from the intestine into the bloodstream and other organs (16). These results would suggest that VVH may not be responsible for the lethality of *V. vulnificus*, but may be a contributor to the tissue damage in pathogenesis. Moreover, other proposed virulence factors characterized to date are not sufficient to explain the acute process of *V. vulnificus* septicemia. The *vvhA* gene is found in most *V. vulnificus* isolates, which was often used as a detecting marker for *V. vulnificus* (17, 18). However, unlike other Vibrio spp. such as *V. cholerae* and *V. parahaemolyticus*, where distinct molecular attributes, such as toxin genes, are normally associated with clinical strains (19, 20). More researchers contend that infections may be driven more by factors associated with host susceptibility than the virulence of *V. vulnificus* (1, 12). Besides *V. vulnificus*, the hemolysins produced by *Vibrio cholerae* and Gram-positive species such as *Streptococcus pneumoniae*, *Streptococcus suis*, *Bacillus Cereus*, have been extensively reviewed (21–23). Like most Gram-negative bacteria, the X-ray crystal structure of VVH remains unknown. However, the mechanisms of pore formation by VVH have been studied in crucial amino acid residues and domains related to the activity of VVH (24–27). Molecular architecture and functional analysis of *V. cholerae* cytolysin (VCC) revealed that VVH has a similar cytolysin domain and a lectin-like domain of VCC (28). However, although these pore-forming cholesterol-dependent cytolysins share structural similarity, they drive divergent cellular outcomes during pathogenesis. In comparison to PFTs in Gram-positive bacteria, more research is needed to clarify the role of VVH in pathogenesis, especially in infections with raw oyster consumption, which can produce rapidly fatal *V. vulnificus* septicemia.

In this review, we explore the features of VVH in its biological activity, regulation of v*vhA* expression, and possible roles in pathogenesis. Future directions in VVH research was also discussed in this review. This in-depth evaluation of the contribution of the VVH to *V. vulnificus* pathogenesis may aid in the development of novel therapies aimed at treating and preventing sepsis in humans.

## Effects of the VVH on Eukaryotic Cells

**Figure 1:**
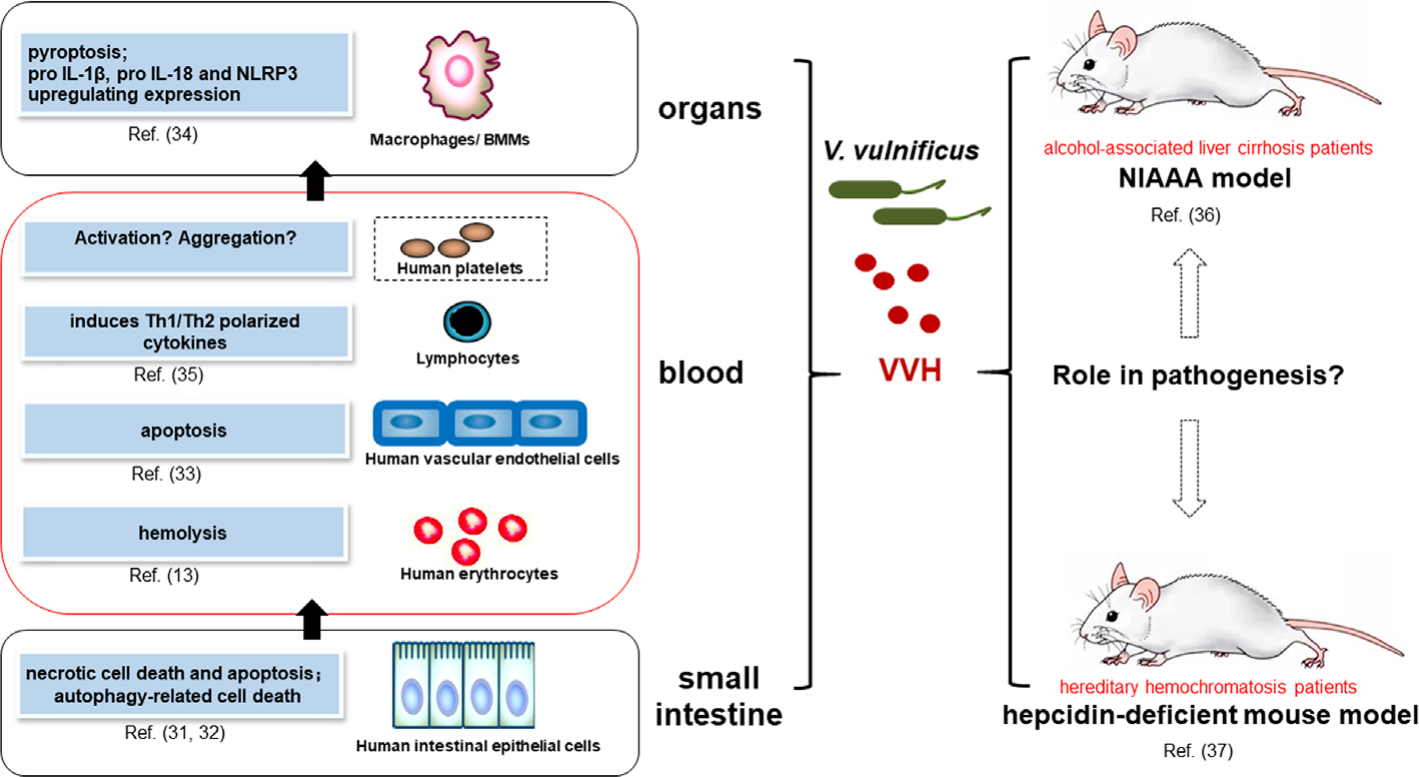
Major activity of VVH’s interactions with host cells and a future perspective of *in vivo* studies involved in pathogenesis. Major activity and mechanism of VVH’s Interactions with host cells mainly focus on intestinal epithelial cells (31, 32), vascular endothelial cells (33), macrophages, (34) and lymphocytes (35), which are possibly involved in bacterial invasion from intestine to blood stream and other organs. However, the effects of VVH on platelets have not been reported. The animal models that mimick human infection will provide a perspective to elucidate the role of VVH in pathogenesis, mainly including the National Institute on Alcohol Abuse and Alcoholism (NIAAA) model (36) and a hepcidin-deficient mouse model (37).

VVH is a 51-kDa water-soluble protein thought to be a member of CDC family of PFT; its hemolytic activity was inhibited by adding cholesterol or divalent cations (29). The VVH causes necrosis, apoptosis, pyroptosis, and lysis in a range of host cell types. First described as a hemolysin, the VVH causes hemolysis of red blood cells in many species, with human erythrocytes being the most susceptible. Although active against erythrocytes from sheep, horses, cows, rabbits, and chickens, the amount of VVH required to cause 50% hemolysis under identical conditions differed between species, suggesting that erythrocyte susceptibility may be closely associated with the binding ability of the VVH and erythrocyte membrane stability (30). *In vitro* studies have illuminated the effects of the VVH in various host cell types, including human epithelial cells, human umbilical vein endothelial cells (HUVECs), mice macrophages and lymphocytes (**Figure 1**), and commonly used cell lines, such as Chinese hamster ovary (CHO) cells (26, 27). However, similar cytotoxic effects have not been reported in human platelets or monocytes.

While *in vitro* studies have revealed the comprehensive effects of the VVH on eukaryotic cells, researchers have also examined the impact of host effectors on the activity of the VVH. Cholesterol is well known for its ability to inactivate the VVH through oligomerization of the toxin monomer (29). However, there are some reports that the VVH recognizes and binds to certain kinds of carbohydrates (38, 39), which suggested that cellular cholesterol is not a receptor for VVH. It may be a trigger factor of conformational changes from membrane bound form to pore-form (39). Moreover, although two studies indicate VVH induces cell death *via* lipid raft-mediated signaling pathway in human intestinal epithelial cells (31, 32), there is no evidence that the VVH localizes at lipid raft so far. One study shows that binding of VVH to target cells does not change by the methyl-beta-cyclodextrin (M*β*CD) treatment (40), and the author subsequently indicates that M*β*CD induces oligomerization of VVH by binding to VVH directly (41).

Besides that, several other factors have also been reported to affect the cytotoxicity of the VVH. Although albumin affects the activity of many different bacterial toxins, Choi et al. reported that neither human serum albumin (HAS) nor bovine serum albumin (BSA) affected *vvhA* transcription or the growth of *V. vulnificus*. However, both HSA and BSA stabilized VVH and delayed its inactivation by oligomerization, thus enhancing VVH activity (42). Blood lipoproteins have also been shown to be an important defense factor against bacterial infection. Park et al. found that low density lipoprotein inactivates the VVH through the oligomerization of the toxin monomer (43). It was widely reported that calcium prevented hemolysis caused by a variety of bacterial hemolysins (44). Jin-Woo Park showed that calcium exerts its major inhibitory effect on *V. vulnificus* cytolysin-induced hemolysis as an osmotic protectant (45). Consequently, trifluoperazine, a calcium-calmodulin antagonist, was found to block the hyperpermeability induced by *V. vulnificus* cytolysin in an *in vitro* modeled endothelium and prevented the deaths of mice (46). Additionally, a recent study showed that melatonin, an endogenous hormone molecule, inhibits apoptotic cell death induced by VVH *via* melatonin receptor 2 coupling with NCF-1 (47). While promising, these results emphasize the fact that we still have much to learn about how the VVH displays its cytotoxic effects *in vivo*, knowledge that will provide important insights into the potential for development of therapeutic strategies and agents to combat *V. vulnificus* infection.

Of special interest is the question of whether the VVH contributes to bacterial invasion from the intestine into the bloodstream and other organs by interacting with host cells. Intestinal epithelial cell death is a host defense response that eliminates damaged cells as well as pathogens to maintain gut homeostasis. However, many bacterial pathogens eventually elicit epithelial cell death and disrupt the gut barrier function to propagate persistent bacterial colonization. A study performed in human intestinal epithelial cells (INT-407) showed that infection with low doses of recombinant VVH protein induces necrotic cell death and apoptosis. The study further demonstrated that (r)VVH induces NF-*κ*B-dependent mitochondrial cell death *via* lipid raft-mediated reactive oxygen species production by the distinct activation of PKCα and ERK/JNK in intestinal epithelial cells (31). Besides VVH has the ability to induce two general modes of cell death, apoptosis and necrosis mentioned above; another study indicated that the VVH induced autophagy-related cell death through the lipid raft-dependent c-Src/NOX signaling pathway in human intestinal epithelial Caco-2 cells. This study further showed that, in an *in vivo* model, VVH increased autophagy activation and paracellular permeabilization in the intestinal epithelium, indicating that VVH plays a pivotal role in the pathogenesis and dissemination of *V. vulnificus via* the upregulation of autophagy, which may provide potential therapeutic targets for strategic modulations of *V. vulnificus* infections (32).

*V. vulnificus* has been shown to produce sufficient VVH in the small intestine to accelerate invasion into the bloodstream (16). Once *V. vulnificus* is in the bloodstream, the VVH interacts with erythrocytes, white blood cells, and vascular endothelial cells. In fact, a recent study has shown that VVH together with MARTX mediates erythrocytes lyses *ex vivo* and, therefore, could contribute to the bacterial growth in human blood that provokes sepsis (13). Researchers observed *in vitro* proliferation of lymphocytes upon re-stimulation of recombinant VVH leukocidin domain (rL/VvhA)-primed splenocytes with formalin-inactivated VVH toxin, while co-expression of T-cell-polarizing cytokines (interferon-*γ*, interleukin (IL)-12, and IL-4) was detected in the cell culture supernatant (35). In an *in vitro* study, the recombinant VVH induces apoptosis in HUVEC cells *via* caspase-9/3-dependent pathway (33). The VVH can also spread to other tissues *via* the bloodstream. Macrophages are large phagocytes found in almost all tissues and play a critical role in increasing inflammation and stimulating the immune system. Claudia Toma et al. indicate that VVH-stimulated NLRP3 Inflammasome activation of bone marrow derived macrophages (BMM), which was induced by TLR and nucleotide-binding oligomerization domain 1/2 ligand-mediated NF-*k*B activation (34). Recently, analysis of VVH-induced inflammation in mice showed that the VVH induces inflammatory responses in RAW264.7 macrophages *via* calcium signaling and causes inflammation *in vivo* (48).

## Regulation of VVH Gene (*vvhA*) Expression

**Figure 2:**
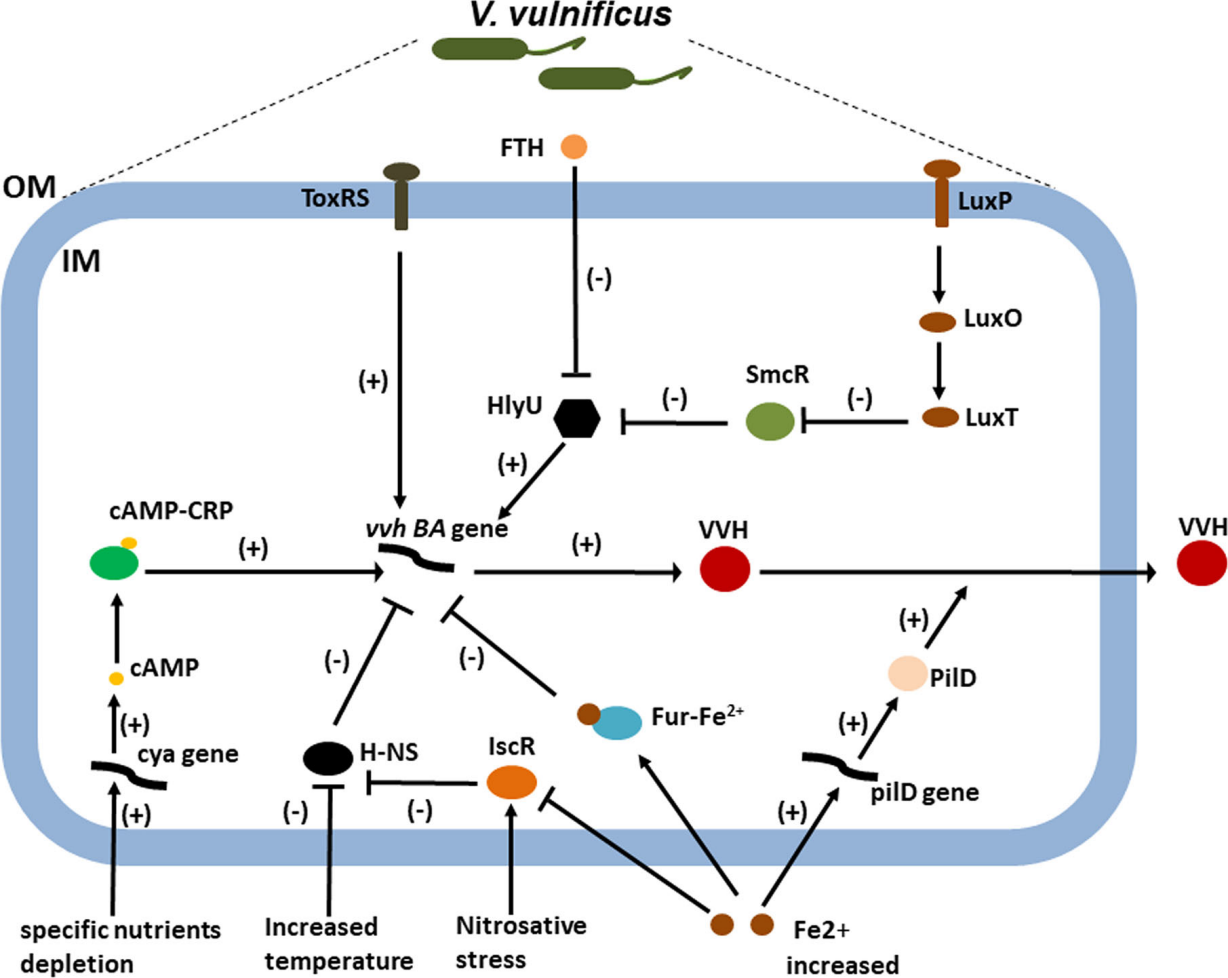
The roles of environmental and host factors and global regulators in the regulation of the VVH expression. CRP activates *vvhBA* transcription in *V. vulnificus* by sensing the depletion of specific nutrients, possibly as a result of increased cAMP levels under glucose starvation (32). Increased iron can repress *vvhA* transcription *via* the ferric uptake regulator (Fur) and IscR (41, 46). However, it increases extracellular VVH secretion through increased transcription of pilD, which encodes PilD, a component responsible for extracellular VVH secretion (41). IscR activates *vvhBA* by relieving H-NS repression by sensing nitrosative stress (46). Meanwhile, a repressive interaction of H-NS would be relieved in response to the increase in temperature (39, 49). LuxO is a central response regulator of the QS circuit in *V. vulnificus*, which negatively regulates *vvhA* expression *via* SmcR and HlyU (42, 43). However, the transmembrane transcriptional activator ToxRS positively regulates the expression of the *vvhA* (47). Taken together, the transcriptional regulators integrate diverse environmental and host signals to collaboratively regulate *vvhA* transcription during the course of infection. Lastly, FTH, an inhibitor target HlyU, was identified to inhibit the transcription of *vvhA* along with that of other HlyU-regulated virulence genes.; OM, outer membrane; IM, inner membrane; FTH, fursultiamine hydrochloride; H-NS, histone-like nucleoid structuring protein; cya, gene encoding adenylate cyclase; cAMP, cyclic AMP; CRP, cAMP receptor protein.

In this review, we will outline the roles of environmental and host factors and global regulators in the regulation of the *vvhA* in terms of expression and transport (**Figure 2**). Cyclic-AMP (cAMP) and bacterial cyclic-AMP receptor proteins (CRPs) represent a classic regulatory system that has been adapted to respond to distinct external and internal signals in many bacteria (50). Hemolysin production in *V. vulnificus* increased after the addition of cAMP but was undetectable in a putative *crp* mutant, suggesting that *vvh* expression is positively regulated by cAMP-CRP in *V. vulnificus* (49). In *V. vulnificus*, cAMP can be produced from adenylate cyclase-encoding gene *cya*. Hemolysin and protease production, motility, and cytotoxicity were all negatively affected by mutation of *cya* (51). CRP activates *vvhBA* transcription in *V. vulnificus* by sensing the depletion of specific nutrients, possibly as a result of increased cAMP levels under glucose starvation (52). In *Escherichia coli*, glucose starvation results in an increase in intracellular cAMP concentrations in response to the altered phosphorylation state of the phosphotransferase system; however, this is difficult to reconcile with observations that the glucose phosphotransferase system remains saturated when intracellular cAMP concentrations increase (53). The regulation of *vvhBA* expression can be more easily examined in the intestine because the availability of free glucose is quite limited. *V. vulnificus* is a ferrophilic bacterium that requires high levels of available iron for growth (12, 13). Although iron can repress *vvhA* transcription *via* the ferric uptake regulator (Fur), it increases extracellular VVH secretion through increased transcription of *pilD*, which encodes PilD, a component of the type II general secretion system responsible for extracellular VVH secretion (14). But there are infection models that suggest that high iron levels (susceptible patients) could also increase *vvhA* transcription (13). So, the regulation of this gene expression should be more complex.

In many pathogenic bacteria, including *V. vulnificus*, quorum sensing (QS) is one of the most important cellular regulatory cascades. QS is responsible for cell–cell communication and is mediated by a small diffusible molecule called autoinducer 2 (AI-2). LuxO is a central response regulator of the QS circuit in *V. vulnificus*, with disruption of *luxO* shown to increase the expression of *smcR*, *crp*, and *luxS*, which encodes the autoinducer 2 synthetase (54). In comparison, SmcR regulates cytotoxicity in *V. vulnificus via* QS signaling by repressing HlyU, which positively regulates *vvhA* expression (55). Temperature is one of the important host parameters regulating the expression of virulence factors in bacteria. The histone-like nucleoid structuring protein (H-NS) global regulator is known to play a crucial role in the expression of temperature-dependent virulence factors. A study on the role of H-NS in temperature-dependent regulation indicated that *hns* expression levels were higher at 26 °C than at 37 °C and that *vvhA* expression and the resulting VVH production were increased following disruption of *hns* (56). Moreover, H-NS, in its role as a *vvhA* repressor, competes with HlyU for binding to the *vvhA* promoter region (57); however, the exact mechanisms of HlyU and H-NS regulation have yet to be fully characterized (56). In addition to cAMP-CRP, Fur, and H-NS, the Fe-S cluster, containing transcriptional regulator IscR, was recently described as an important regulator of *V. vulnificus* virulence in host environments. IscR activates the *vvhBA* operon in response to nitrosative stress and iron starvation, thereby aiding successful host infection (58). Lastly, transmembrane transcriptional activator ToxRS, a homolog of the *V. cholerae* ToxRS transmembrane virulence regulator, may also positively regulate the expression of the *vvhA* (59). In summary, recognition of the subtle regulation of v*vhA* gene expression and hemolysin delivery by *V. vulnificus* has furthered our understanding of how the VVH contributes to disease pathogenesis.

The complicated *vvhA* regulatory system that emerges from this data suggests that inhibition of global regulators may be a promising approach for the development of alternatives to antibiotic treatment. Recently, an inhibitor-screening reporter platform was used to target HlyU, a master virulence factor transcriptional regulator in *V. vulnificus*. The study identified a small molecule called fursultiamine hydrochloride that inhibited the transcription of *vvhA* along with that of other HlyU-regulated virulence genes. Fursultiamine hydrochloride therefore has the potential to inhibit the pathogenesis of *V. vulnificus* without inducing antimicrobial resistance (60).

## The Role of the VVH in Disease and Pathogenesis

*V. vulnificus* most commonly causes severe gastroenteritis following the consumption of contaminated raw seafood, with sepsis infection mortality rates of 50% (12). Moreover, because *V. vulnificus* is responsible for >95% of seafood-associated infection deaths in the United States (4), a significant number of studies have focused on the effects of the VVH on human intestinal epithelial cells mentioned above. In addition, small intestine-associated host factors together with mouse models have been used to investigate the role of the VVH in pathogenesis. The human intestine usually secretes cationic antimicrobial peptides to prevent pathogen colonization, with Paneth cells in the small intestine secreting antimicrobial molecule alpha-defensin 5 (HD-5). However, while HD-5 inactivated the *Vibrio mimicus* hemolysin, it had no effect on VVH. The inability of *V. mimicus* to penetrate the small intestinal epithelium suggests that the cytolytic activity of the *V. mimicus* hemolysin is abolished by HD-5 (61). In contrast, *V. vulnificus* causes intestinal tissue damage and inflammation, which then promotes dissemination of the pathogen from the small intestine into the bloodstream and other organs in infected mice (6, 7). Notably, the small intestine is recognized as the site of the most severe tissue necrosis in humans based on autopsy results from *V. vulnificus*-infected patients (62). Indeed, VVH and MARTX are the two *V. vulnificus* virulence factors associated with both enhanced growth *in vivo* and necrosis of tissue in the small intestine, followed by dissemination into the bloodstream and other tissues. In the absence of these two secreted factors, *V. vulnificus* is unable to cause intestinal infection in mice (16).

*V. vulnificus* also causes primary septicemia in patients with underlying liver disease or who are immunocompromised (63). Patients with septicemia tend to die of hypovolemic shock complicated by multi-organ failure. A study in rats found that the VVH dilates the thoracic aorta by activating guanylate cyclase, causing hypotension *in vivo* and vasodilatation *in vitro* (64, 65). *V. vulnificus* can be spreading from the intestine to bloodstream. To survive and proliferate in blood, *V. vulnificus* requires to overcome the innate immune defenses, including complement-mediated phagocytosis. Recently, capsular polysaccharide and Flp (fimbrial low-molecular-weight protein) pili are reported to play critical roles in evasion of the host innate immune system by resistance to complement-mediated killing (66, 67). Although an earlier work showed that virulent isolates produced high titers of hemolysin, were resistant to inactivation by serum complement (68), further information is needed to uncover the mechanism of VVH-mediated evasion of complement killing, which may help us to better understand the basis of the *V. vulnificus* infection process in human blood. Being at the crossroads between the immune system, clotting cascade, and endothelial cells, platelets seem to be an appealing central mediator and possible therapeutic target for sepsis (69–71). The mechanism of bacterial-induced platelet activation by pore-forming toxins has been well characterized in other Gram-positive bacteria (72). However, despite the significant fatality rate associated with *V. vulnificus*-induced sepsis, the interaction between the VVH and platelets is not clear. Because the CDC of Vibrio spp. share structural similarity (28), it is possible that VVH represents a critical molecule of Vibrio spp. involved in pathogenesis by interacting with platelets. Linked to this, efforts should be focused on the mechanisms of VVH-induced platelet activation for future work.

## Conclusions and Future Perspective

Cholesterol-dependent cytolysins are a diverse group of proteins that differ between bacterial species. However, it is these differences that have informed much of our understanding of the biological activities of the proteins, as well as their role in pathogenesis. Despite this insight, further studies are needed to determine the structure–function relationships of the VVH. Functionally, the major roles of the VVH are to induce cytotoxicity by binding to the cellular membrane to form pores and activating the host inflammatory response. These functions, along with the subtle regulation of VVH gene expression and other potentially unrecognized activities, contribute to the pathogenesis of *V. vulnificus* disease. Although the host response to the VVH involves lipid raft-dependent signaling pathway-mediated cell death, it is likely that other mechanisms may also be involved in the host response to the VVH.

*V. vulnificus* infection can result in severe disease. In fact, most cases occur in patients with underlying conditions resulting in hereditary hemochromatosis, primarily alcohol-associated liver cirrhosis or immuno-compromised males, but it does not cause severe illness in healthy individuals (73). Although there have been many studies on the effects of the VVH on eukaryotic cells *in vitro*, few animal models that mimick human infection were used to elucidate the role of VVH in pathogenesis. As a result, we still have much to learn about how this toxin contributes to disease pathogenesis *in vivo* (**Figure 1**). An interesting study found that hepcidin has a critical role in host defense against *V. vulnificus* by inducing reactive hypoferremia during early phases of infection (74). Hepcidin is a 25 amino acid peptide secreted by hepatocytes. Hereditary hemochromatosis is caused by deficiency of the iron-regulatory hormone hepcidin (75). Therefore, a hepcidin-deficient mouse model of severe hemochromatosis (37) could be considered for the future work about the role of VVH in the lethal infections by *V. vulnificus*, a siderophilic bacterium. Additionally, the National Institute on Alcohol Abuse and Alcoholism (NIAAA) model is a mouse model of chronic and binge ethanol feeding, which mimics acute-on-chronic alcoholic liver injury in patients (36). This simple model will be very useful for the study of the function of VVH *in vivo*, and the underlying mechanisms that contribute to acute infections by *V. vulnificus* in liver disease patient.

## Author Contributions

YY contributed to the research of the literature and the writing and revision of the manuscript. ZF and JW contributed to the revision of the manuscript. All authors contributed to the article and approved the submitted version.

## Conflict of Interest

The authors declare that the research was conducted in the absence of any commercial or financial relationships that could be construed as a potential conflict of interest.
